# 

**Published:** 1781-05

**Authors:** 


### PROMOTIONS.

Dr. Henry Revell Reynolds, and Dr. Thomas Bowdler, physicians in London, to be Fellows of the Royal Society.—Dr. Martin Wall to be reader in chemistry at Oxford.—*April* 28. Mr. Valentine Jones to be surgeon to the first troop of horse guards, in the room of Mr. Robert Sinclair.—*May* 26. Dr. Thomas Clerk, member of the College of Physicians, London, to be physician to the forces in North America, under the command of Sir Henry Clinton.—Mr. Patrick trick Connor, late aſſiſtant ſurgeon to the hoſpital at St. Vincent's, to be ſurgeon to the hoſpital in the Leeward Iſlands.

---

### DEATHS.

*March* 11.—At Reading in Berkſhire, Mr. Palmer, apothecary, aged 63 years.

24.—At Watton in Norfolk, Mr. Robinſon, ſurgeon.

26.—At his ſeat at Huntingdon in Scotland, George Gray, M. D. formerly phyſician to the factory at Calcutta.

*April* 7.—In Lamb-ſtreet, Spital-fields, London, Mr. Peter Moſes Chaſtelier, ſurgeon.

12.—At Millthrop in Weſtmoreland, Mr. Francis Herd, ſurgeon.

21. At Morpeth, Northumberland, Mr. Reed, ſurgeon.

28.—At Newcaſtle upon Tyne, Mr. John Carr, ſurgeon.

*May* 9.—At Pendleton, Lancaſhire, aged 54 years, Mr. Thomas Diggles, formerly a ſurgeon and apothecary at Mancheſter.

### SECTION IV.

### MONTHLY CATALOGUE.

I. *Hints on Diseases that are not cured; addressed to the Faculty only.* 4to. *Murray*, London, 1781. 46 pages. 1s. 6d.

THIS pamphlet is written in an agreeable manner, and seemingly with a view to draw the attention of medical practitioners to certain diseases usually deemed incurable, rather than to propose any new modes of treatment in such cases. The author has confined his reflections chiefly to consumption and gout. "At a period, "says he, which teems with valuable treatises, "on almost every other disease; and when we "are threatened with an inundation of simple "facts from every quarter, the number of vo-"lumes on pulmonary phthisis begins to dimi-"nish, and the gouty patient is left to tell his "own story."

As a means of improving our practice in cases of phthisis, he proposes the institution of an infirmary, which might consist of one or more houses, in a dry, airy, but not very exposed situation, for the reception of poor consumptive

ſumptive patients. "If one of them, he ob-

"ſerves, were ſituated near the ſea, it would

"afford the probable advantages of ſea air, and

"an opportunity of ſailing, an exerciſe ſo highly

"extolled, that we have ſome reaſon to think

"on this confined ſcale, it might prove no deſ-

"picable auxiliary. The relative ſituation of a

"lime-kiln, or any manufactory which occa-

"ſions copious exhalations, may determine the

"choice of other receptacles. Let each of

"them be ſpacious; yet never contain more

"patients than one phyſician and one apothe-

"cary may well take the charge of: the for-

"mer ſhould attend at leaſt three times a week,

"and keep an exact regiſter of every caſe that

"falls under his inſpection. His ſagacity muſt

"direct him further; but he will, I believe,

"ſee the propriety of proceeding on the plan

"of ſimple, and cautious medicinal treatment

"already recommended. He will firſt apply

"himſelf to learn the beſt regimen, and how

"far it alone may be relied on; and when he

"ſhall have occaſion to call in remedies to his

"aid, he will give them one by one a fair trial,

"and thus he will be able to aſſign to each its

"reſpective degree of merit."

In

In gouty cases he proposes, that the books which make mention of anti-arthritic medicines should be consulted, and that all such as are not absurd or evidently insignificant, should have a fair trial, and their effects be carefully attended to.

2. Outlines of the theory and cure of fever, upon plain and rational principles, by *John Aitken*, Fellow of the Royal College of Surgeons, of the Royal Medical Society, one of the Surgeons of the Royal Infirmary, and lecturer on anatomy, surgery, and chemistry in Edinburgh, 8vo. *Cadell*, London, 1781. 88 pages.

The first thing that attracted our notice, on opening this little production, was the following dedication to COMMON SENSE: "Thou venera-"ble arbiter of science and of human conduct, "universally to be cultivated and adored! How "seldom do the sons of Æsculapius bend as "humble votaries at thy shrine! Many of them "have lamentably secluded themselves from thy "irradiating influence; some bewildered by "numberless mounds of mud, their own crea-"tion; some confounded by artificial clouds "and darkness; and some, strange to tell! have "fatally deviated from the path leading to thy "temple, because it is patent and obvious. Of-

"tener

"tener have they idolatrously become the steady "devotees of chaos, thy potent and gloomy "rival, bending to the fictions of their mon-"strously teeming imaginations.

"If ever thy feeblest ray has in any degree "illumined my mind, deign to accept this dedi-"cation of *Outlines of the Theory and Cure of* "*Fever*, as a respectful oblation from thy hum-"blest admirer, THE AUTHOR."

Mr. Aitken supposes a *plastic power* to be inherent in animal as well as vegetable bodies, the operation of which he names *the plastic or healing process*, because it tends directly to the cure of disease. He thinks that the art of physic possesses little or no immediate influence over this principle, and is only capable of assisting or co-operating indirectly with it. He rejects the use of blisters in the cure of fever, because "they "are manifestly deranging powers, and therefore "ill suited to promote or second the plastic "process."

3. Elements of anatomy and the animal oeconomy. The second edition, with considerable alterations and additions. By *Samuel Foart Simmons*, M. D. F. R. S. &c. 8vo. *Walker*, London, 1781. 418 pages. 5s. *in boards.*

With

With the firſt edition of this uſeful compendium, our anatomical readers are doubtleſs well acquainted. In the preſent edition it is greatly enlarged and improved. Amongſt other additions Dr. Simmons has given a table of the muſcles, deſcribing the origin, inſertion, and uſe of each; he has likewiſe introduced a more copious deſcription of the brain than is to be met with in the firſt impreſſion, and as he has availed himſelf of all the lateſt improvements in anatomy, his work cannot but prove highly acceptable to medical ſtudents.

4. A treatiſe on the gonorrhœa; to which is added a critical enquiry into the different methods of adminiſtering mercury. Intended as a ſupplement to a former work, intitled, A new and eaſy method of cure by the introduction of mercury into the ſyſtem through the orifices of the abſorbent veſſels on the inſide of the mouth. By *Peter Clare*, ſurgeon, ſmall 8vo. *Cadell*, London, 1781. 60 pages.

The greateſt part of this pamphlet conſiſts of quotations from Boerhaave, Mead, Woodward, Alſton, Geo. Fordyce, Smith, and Plenk, but chiefly from the three latter. Mr. Clare avows himſelf a convert to the uſe of cold vitriolic injections in gonorrhœa, provided at the ſame time fame time a grain of mercurial powder is daily applied to the cuticular furface of the lips. Mercurial preparations he has generally found too irritating. His critical inquiry feems intended to prove, that the rubbing calomel on the infide of the mouth is the fafeft and moft effectual method of introducing mercury into the fyftem.

5. Recherches analytiques fur les eaux de la fontaine minerale de Jaleyrac dans la Haute Auvergne, avec un precis des maladies où ces eaux peuvent être utiles, des cas où elles pourroient être prejudiciables, et la maniere de les prendre avec fuccés. Par M. *Pierre André de la Rouſſerie*, D. M. Medecin a Neuvie en Limoufin. *i. e.* Analytical inquiries concerning the mineral waters at Jaleyrac in Upper Auvergne, with an account of the difeafes in which they may be ufeful, of the cafes in which they might prove hurtful, and of the method of taking them with fuccefs. By *Peter Andrew de la Rouſſerie*, M. D. Phyfician at Neuvie in Limoufin. 12mo. Tulle. 1780. 21 pages.

6. Differtation fur le charbon malin de la Bourgogne, où la puftule maligne : ouvrage couronné par l'academie des fciences, arts & belles lettres de Dijon, le 14 Fevrier, 1780,

*i. e.* A differtation on the carbuncle or malignant puftule of Burgundy, being the work crowned by the academy of fciences, arts, and belles lettres at Dijon, Feb. 14, 1780. 8vo. Dijon, 1780.

7. Compendio di notizie intereffanti circa il veneno di rabbiofi animali. *i. e.* A compendium of interefting remarks on the poifon of mad animals. By *Felix Afti*, M. D. 4to. Mantua, 1779, 110 pages.

This compilation would have been more ufeful, if the author had availed himfelf of the refearches of feveral modern writers, with whofe works he feems to be unacquainted. Towards the clofe of the pamphlet we meet with a few cafes, from which we learn, that feveral perfons at Pomponefco ate part of a hog that had been bit by a mad dog, without experiencing any ill effects from it. The author likewife informs us, that the flefh of an ox under fimilar circumftances proved equally innocent. He fpeaks of a mad wolf that bit a great number of perfons in the neighbourhood of Lonato, moft of whom perifhed. One woman, however, was cured by the internal and external ufe of mercury; and another, who was delivered of a child the day after the accident, efcaped by taking Bellini's powder, powder, which is a mixture of cantharides and pepper. It does not appear, however, that either of these two patients had received the infection.

8. Eloge de M. Maret, Maitre en Chirurgie a Dijon, ancien chirurgien major de l'hospital general, pensionnaire veteran de l'academie des sciences, arts et belles lettres de la meme ville, par *M. Maret*, Docteur en Medecine, secretaire perpetuel de l'academie: lû dans la seance du 17 Decembre 1780. *i. e.* The Elogy of M. Maret, surgeon at Dijon, formerly surgeon to the General Hospital, and member of the academy of sciences, &c. by *M. Maret*, M. D. secretary to the academy. Read at the meeting on the 17th of December 1780. 8vo. Dijon. 32 pages.

John Philibert Maret, the subject of this little work, was born at Dijon, November 8, 1705. He studied surgery first under his father at Dijon, and afterwards at Rome and Paris. He died October the 14th 1780, unmarried, leaving the author and seven other nephews and nieces to regret his loss. He was the author of three dissertations presented to the academy of Dijon. In one of these he attempts to prove, that in dying persons hearing is the last fenfe extinguifhed. His fecond paper was on amputation, and the third on lithotomy.

9. Detail des fuccés de l'etabliffement que la ville de Paris a fait en faveur des perfonnes noyées, et qui a été adopté dans diverfes provinces de France. Par *M. Pia*, ancien echevin de la ville de Paris, & chevalier de l'ordre du roi, &c. *i. e.* An account of the fuccefs of an eftablifhment inftituted by the city of Paris in favour of drowned perfons, and which has been adopted in feveral provinces of France. By *M. Pia*, formerly fheriff of the city of Paris, and knight of the king's order. 8vo. Paris. 3d edition, 1780.

10. Phyfica hominis fani, feu explicatio functionum corporis humani, auctore *Nicolao Jadelot* regis confiliario et medico, anatomiæ & phyfiologiæ in univerfitate Nanceiana profeffore, academiæ regiæ fcientiarum et artium Nanceianæ ac focietatis regiæ medicæ Parifinæ focio; nofocomii Nanceiani a Sancto Carolo dicti medico. 8vo. Nanceii, 1779, 249 pages.

An inaccurate compendium of phyfiology, written in an obfcure ftyle.

11. *Alberti von Haller* primæ lineæ phyfiologiæ auctæ ab *H. A. Wrifberg*, M. D. anatomes et et artis obstetric. prof. ordin. Gotting. 8vo. Göttingen, 1780, 550 pages.

This excellent work is greatly improved by the present editor's judicious notes.

12. Einleitung in die Wissenschaft der rohen und einfachen Arzneymittel, &c. Von *J. G. Gleditsch*, D. A. D. &c. *i. e.* An introduction to the knowledge of simple medicines upon physico-chemical and medico-practical principles. By *J. G. Gleditsch*, M. D. and professor of botany at Berlin. 8vo. Berlin, 1779, 2 vol.

In this work the medicinal simples are described with great precision, and the characteristics of their goodness or adulteration carefully ascertained.

13. *J. F. Van Berkley*'s Naturgeschichte von Holland, &c. *i. e.* The natural history of Holland, by *J. F. Van Berkley*. Translated from the Dutch. With copper plates. Vol. I. 8vo. Leipsic, 1779, 314 pages.

This writer appears to be unacquainted with many late discoveries in natural history. He confines himself chiefly to a description of the atmosphere, soil, rivers, &c. of Holland, and passes slightly over its animals, vegetables, and minerals. In his arrangement he follows M. M. Buffon and D'Aubenton.

14. Theoriæ

14. Theoriæ phænomenorum electricorum, quæ, feu electricitatis ex redundante corpore in deficiens trajectu, quæ fola atmofpheræ electricæ actione gignuntur. Auctore *J. de Herbert*, prof. philofophiæ naturalis. 8vo. Vienna, 1779, 246 pages, with copper plates.

This very ingenious performance, contains a great number of new and interefting experiments in electricity. In his feventh chapter, fpeaking of the effects of electricity upon organized bodies, the author mentions an experiment on two boys by weighing them in a pair of fcales, one of which was fufpended by filk; and he affures us, that the boy in the latter fcale, after being ftrongly electrified, became much lighter than the other. Having procured recent portions of a nerve, mufcle and artery, he found the firft to be as good a conductor as metal, while the mufcle was lefs fo, and the artery did not conduct at all. But the next day the nerve, when dry, was as incapable of tranfmitting electricity, as the artery. Hence our author concludes, that its conducting powers depended on the exiftence of a nervous fluid. In noticing the medicinal effects of electricity, our author tells us he has often obferved, that after the cure of obftinate difeafes of long ftanding, ftanding, fuch as palfies, for inftance, by electricity, the removal of the original complaint has been fucceeded by rheumatic fevers, catarrhs and diarrhœas.

15. Abhandlungen zur naturgefchichte, phyfik und oekonomie, aus den Philofophifchen Tranfactionen und Samlungen, vom erften Bande angefangen gefamelt und mit einigen Anmerkungen überfetzt erfter Band. *i. e.* Effays on natural hiftory, natural philofophy, and rural œconomy, extracted from the Philofophical Tranfactions, with notes. Vol. I. 4to. Leipfic, 1779, 292 pages.

The German reader is indebted for this ufeful compilation to Mr. Lefke, profeffor of natural hiftory at Leipfic, who has enriched it with a great number of ufeful notes.

16. *H. A. Wrifberg*, M. D. et anatomes profefforis differtatio de præternaturali et raro inteftini recti cum vefica urinaria coalitu et inde pendente ani defectu obfervationibus anatomicis fuperftructa. Cum figuris. 4to. Göttingen, 1779.

The fubject of this differtation was a child, who died eight days after its birth. The appearances on diffection are well exhibited in the engravings that accompany the work.

17. *H. A. Wrifberg*, M. D. &c. Obfervationes anatomicæ de tefticulorum ex abdomine in fcrotum defcenfu, ad illuftrandam in chirurgiâ de herniis congenitis utriufque fexus doctrinam, cum figuris. 4to. Göttingen, 1779.

The intention of our author is to fhew, how the defcent of the tefticles may give rife to an hernia congenita. He firft quotes the obfervations of different modern anatomical writers, and then adds the refult of his own inquiries on this fubject. He finds, that the teftes do not defcend fo low as the abdominal rings till the fixth or feventh month, and that the fcrotum till that time is fmall and empty. He obferves, that the tefticles while in the abdomen are covered only by a minute procefs of the peritonæum, and afford no appearance of a tunica vaginalis. In his diffections, he has feveral times found a part of the omentum or inteftines adhering to the tefticle in the abdomen of the fœtus, and when this happens, an hernia congenita will be likely to take place. The fame thing, he adds, will occur, when the peritonæum in its courfe over the feminal veffels to the mefentery, fends off a minute procefs to the ileum or cæcum, and by means of it draws down the inteftines on the right fide, which is the common feat of the hernia congenita.

